# The Close Relation Existing between, and Probable Identity of, Scarlatina and Diphtheria

**Published:** 1883-03

**Authors:** 


### THE CLOSE RELATION EXISTING BETWEEN, AND PROBABLE IDENTITY OF, SCARLATINA AND DIPHTHERIA.

The occult cause of disease, malaria, miasm, or whatever you please to call the poison, and how many different and modified types of diseases you will notice in that relation, viz., the different forms of intermittent, remittent and typo-malarial fever (the latter a misnomer), etc., etc., so different in their behavior and general symptoms that one would hardly think they had a common origin. But still the doctor will look wise, for he has been taught no better, and tell the patients —none of them showing the same symptoms in common—that they are all suffering from that mysterious, occult something called malaria, and probably he is correct. Look if you please at the more particularly infectious diseases, those that are not only spread by absolute contact, but by emanations from the body; the secretions, etc., are caught up in the air, and enter the circulation by absorption or inhalation, as germs, spores or bacilli, and thereby cause disease, and note the various forms and diseased conditions manifest in the different patients suffering from the same disease, with almost completely dissimilar symptoms, all supposed to have a common cause producing them, and how do doctors account for these seemingly contradictory manifestations? Merely the cases are modified by extraneous forces, the patient's surroundings and his physical condition. I need not point out in a particular manner these differences, because all present, if they will reflect one moment, will at once admit the force of the statements made; and that they have all, at some period of their professional lives, felt doubts as to the correctness of their forced conclusions. But if we simplify our theories, if we throw aside the mysterious somethings—seen by the curious—"sometimes figments of the brain," the germs, spores, etc., which are supposed to be specific, and are no doubt thought to be so in the original investigator's office, but occult in practice, and which are left to the common honest plodding practitioner in his daily experience at the bedside, to approve or reject, for he cares but little about theories, but treats these diseases with good success, because he treats conditions and symptoms rather than names, and adopt the more rational theory, more in accordance with facts, that, *per se*, there are but few specific generic diseases, or groups of disease, and as in the vegetable kingdom the classes and genera are few, the varieties vast, so in the pathological field, then we shall more easily and fully comprehend the true causes of disease, and treat them more rationally and successfully. It requires no great reach of imagination or elasticity of our professional conscience to acknowledge the truth, that we are all more or less in doubt and at sea in reference to the real causation of disease, and that the theories piled up, plus all our doubts, multiplied by what we do not know, equals an amount of professsional, negative and empirical truth, which should be a shame to the votaries of an exact science to acknowledge.

You are all familiar with the general characteristics, the course, etc., of the two diseases, so called, scarlatina and diphtheria. You know the general symptoms present in each, you can trace their analogies, you have learned their pathology, if not their etiology. The parts affected—the throat, the lymphatic glands, the skin, the mucous membrane and the general constitutional disturbance of the system. Now, keep all of these associated symptoms in your minds, the co-relation of facts and conditions in both, and can you not see that both diseases present many clinical phases common to each? I cannot, of course, enter into a detailed account of the varied symptoms and full clinical history which they present in their progress, or fully elaborate the subject, because it is too vast, wide and deep, for the time given me and my poor capacity to develop. But it shall be my province to present such facts as I can, the result of reading, observation and personal experience as will at least direct your attention to the subject under consideration.

During the year 1864 we had in our city an epidemic of diphtheria, so called by some practitioners; others claimed it was scarlatina. I then held to the belief that we had both varieties and forms of disease depending upon a common cause, because we had both the symptoms and pathological conditions common to the manifestations of both forms of disease during the progress of the epidemic; sore throat with exudative deposits or false membrane, enlarged lymphatic glands, etc., with the characteristic eruption and without the eruption. We had in the same families the various sequelæ common to both forms of disease, viz., paralysis, acute rheumatism, dropsy and croup. Notably in one family, of which I have notes of the cases, two boys and two girls; one boy and girl had eruption on skin well marked and characteristic; the others had none; all had throat complication. The youngest died, from croup and two of the others had sequelæ; one had dropsy depending upon kidney complication, another had partial paralysis, while the remaining one had no sequelæ.

In Sir Thomas Watson's work on "Practice," etc., page 996, we find the following explicit statement in strong language: "The two striking and important features of the disease are the affection of the throat and the affection of the skin. They may both be well marked or only one of them may be well marked; and this circumstance has led nosologists to divide one and the same complaint into two different maladies, to which Cullen and others have assigned the respective names of cynanche maligna and scarlatina. When in the early part of the course I was treating of the diseases of the throat, I purposely omitted the cynanche maligna, because that is only a name for a particular form of scarlet fever. If you look at Cullen's definitions of these complaints, you will see how much alike they are. They both specify inflammation of the fauces, a cutaneous rash and fever. The truth is, that these two kinds of diseases both spring from the same contagious poison. "The malignant sore throat" (the same as putrid sore throat) "may be caught from a patient who has mild scarlet fever, and mild scarlet fever may, in like manner, be contracted trom one who is laboring under malignant sore throat. The two forms graduate insensibly in different cases toward each other; and it would be impossible, even if it were desirable, to draw any strict line of separation between them." Further, on page 998, he holds: "As the disease proceeds, the throat becomes foul and sloughy, the parotid and submaxillary glands swell, sometimes enormously, and fever is lighted up afresh. The acrid matter furnished by the ulcerating and gangrenous throat—" "And this swelling of the parotids and neighboring glands is evidently caused by the absorption of the irritating and poisonous matter from the ulcerated and gangrenous throat."

Now, when we reflect that at the time the above lucid and graphic description was given of the real pathological condition of the throat, during the progress of scarlatina maligna, or putrid sore throat, since named diphtheria, you will at once see that the symptoms and diseased condition delineated could be none other than our present diphtheria, which was clearly claimed by the authority quoted as only a modified form of the cardinal disease, scarlatina. It seems that most authorities, even Professor Flint, after laboring to draw a line of distinction between the two forms of disease, still associate them together, and are in doubt as to the relation which they sustain to each other, whether distinct and cardinal, or whether the one may not be the result of the other. On page 817 you will find this language: "This boy had recently had diphtheria, the exudation in the fauces being abundant. There had been no eruption. After his convalescence, a sister was attacked with scarlatina, the rash being abundant. The boy had the characteristic sequels of both diphtheria and scarlatina, and there was ground for the suspicion that he communicated the latter affection to his sister." Not to be captious, probably if there had been another case in the same family—another child sick—the sister would have communicated diphtheria back again to the other member of the family. Interchangably, like the "shuttle cock," the disease could have been thrown "backwards and forwards" to suit the caprice of the observer.

Now I hold that the old fashioned purtid sore throat, pseudo-membranous pharygitis and diphtheria—rechristened by Dr. Bland, of New York, and more especially by M. Bretonneau, of France, in 1821, are one and the same disease, and that the disease has prevailed in epidemic form, at different times, and such times described by different writers, using different names, but mainly agreeing with each other in the symptoms and general clinical history of the disease, and that such clinical history given of diphtheria and scarlatina maligna, the parts affected, the general characteristics and course of the diseases would seem to be something more than a mere coincidence, and that probably and primarily they have a common parentage. Read Ortel on diphtheria and Thomas on scarlatina maligna, and note the similarity in the main features and clinical behavior of the two diseases, laying aside mere theories, long drawn out, detailing the results of many experiments of microscopic observations, the kind of spores seen, how they behave and what they do. After all, clinically you can see such a strong analogy manifested in each grave form of the diseases, that mere theory as to their separate causation will not satisfy the common practitioner. And it would seem just as reasonable and just as practicable to maintain their common causation as to adopt the idea of the duality of their origin. For it seems to me that the recognition of a radical difference between diseases which possess strong points of resemblance and analogy, is a very delicate matter, and that the arguments in favor of such separation and division of causes, in reference to two grave diseases so similar in their character and analogous in their results, and demanding so nearly the same management and treatment. For certainly rather than amplify our nosology it would seem wisdom to curtail it. But so it is, that every observer who discovers a spore, or a bacillus must name a disease, and hence they multiply.

We now come to the eloquent and sagacious Trousseau, the great clinical teacher, who from his long experience, ample opportunities for clinical observation and research, would seem to be pretty good authority upon the subject under consideration. Vol. 1, page 149, in treating of scarlatina, says : "Has diphtheria supervened to complicate the scarlatina, and divert it from its proper course? The symptoms bear so strong a resemblance to the terrible forms of that frightful disease which carry off both adults and children before the affection has extended to the larynx, the false membrane still remaining localized in the nasal fossæ, ears and throat, the symptoms so much resembling the rapidly fatal forms of diphtheria that one is induced to believe that the case is no longer one of scarlatina, but that the other dreadful scourge has come to destroy the patient." "During my period of service at the children's hospital, I so often found such an extraordinary identity between the sore throat of malignant diphtheria that I became shaken in my opinion." Such are the conclusions drawn and opinions held by one who had great opportunities to observe the diseases in all their phases, and we see that he even expresses doubts as to the duality in causation of the diseases under consideration. To me it seems obviously absurd, and contumacious to strive to separate, and discuss propositions in reference to diseases where one seems to be, and is admitted to be, the analogue of the other. Certainly there is no reason why, when there are no improved suggestions as to the treatment resulting from the maintenance of the dual cardinal distinction in the causes of such diseases, put forth by different authors.—*Extracts from article by Dr. Hurd, in Detroit Lancet, February*, 1883.

